# Snake Bite in South Asia: A Review

**DOI:** 10.1371/journal.pntd.0000603

**Published:** 2010-01-26

**Authors:** Emilie Alirol, Sanjib Kumar Sharma, Himmatrao Saluba Bawaskar, Ulrich Kuch, François Chappuis

**Affiliations:** 1 Division of International and Humanitarian Medicine, Geneva University Hospitals, Geneva, Switzerland; 2 B. P. Koirala Institute of Health Sciences, Dharan, Nepal; 3 Bawaskar Hospital and Research Centre, Raigad, India; 4 Biodiversity and Climate Research Centre, Frankfurt am Main, Germany; Faculty of Medicine, University of Kelaniya, Sri Lanka

## Abstract

Snake bite is one of the most neglected public health issues in poor rural communities living in the tropics. Because of serious misreporting, the true worldwide burden of snake bite is not known. South Asia is the world's most heavily affected region, due to its high population density, widespread agricultural activities, numerous venomous snake species and lack of functional snake bite control programs. Despite increasing knowledge of snake venoms' composition and mode of action, good understanding of clinical features of envenoming and sufficient production of antivenom by Indian manufacturers, snake bite management remains unsatisfactory in this region. Field diagnostic tests for snake species identification do not exist and treatment mainly relies on the administration of antivenoms that do not cover all of the important venomous snakes of the region. Care-givers need better training and supervision, and national guidelines should be fed by evidence-based data generated by well-designed research studies. Poorly informed rural populations often apply inappropriate first-aid measures and vital time is lost before the victim is transported to a treatment centre, where cost of treatment can constitute an additional hurdle. The deficiency of snake bite management in South Asia is multi-causal and requires joint collaborative efforts from researchers, antivenom manufacturers, policy makers, public health authorities and international funders.

## Introduction

Since ancient times, snakes have been worshipped, feared, or loathed in South Asia. Cobras appear in many tales and myths and are regarded as sacred by both Hindus and Buddhists. Unfortunately, snakes remain a painful reality in the daily life of millions of villagers in this region. Indeed, although antivenom is produced in sufficient quantities by several public and private manufacturers, most snake bite victims don't have access to quality care, and in many countries, both morbidity and mortality due to snake bites are high. The neglected status of snake bite envenoming has recently been challenged [1] but as outlined below, apart from the production of antivenom, snake bite envenoming in South Asia shares all the characteristics of a neglected tropical disease. This review aims at summarizing and discussing the epidemiology, clinical features, diagnosis, and treatment of snake bite envenoming in South Asia (Bangladesh, Bhutan, India, Nepal, Pakistan, and Sri Lanka).

## Methodology

Articles were identified by searching Medline through PubMed using various combinations of terms including “snake,” “snake bite,” “envenoming,” and “venom.” Research papers and case reports from Bangladesh, Bhutan, India, Nepal, Pakistan, and Sri Lanka were retrieved, as were significant papers from other Asian countries. Additional articles were obtained by citation tracking of review and original articles. The review also drew on conference proceedings and original research conducted by the authors.

## Epidemiology

An accurate measure of the global burden of snakebite envenoming remains elusive despite several attempts to estimate it and, apart from a few countries, reliable figures on incidence, morbidity, and mortality are scarce [2]–[4]. South Asia is by far the most affected region [2],[4]. India has the highest number of deaths due to snake bites in the world with 35,000–50,000 people dying per year according to World Health Organization (WHO) direst estimates [2],[4]. In Pakistan, 40,000 bites are reported annually, which result in up to 8,200 fatalities [4],[5]. In Nepal, more than 20,000 cases of envenoming occur each year, with 1,000 recorded deaths [6]. In Sri Lanka, around 33,000 envenomed snake bite victims are reported annually from government hospitals [4],[7]. A postal survey conducted in 21 of the 65 administrative districts of Bangladesh estimated an annual incidence of 4.3 per 100,000 population and a case fatality of 20% [8]. However, existing epidemiological data remain fragmented and the true impact of snake bites is very likely to be underestimated. Surveys in rural Sri Lanka showed that hospital data record less then half of the deaths due to snakebite [9]–[11]. In Nepal, a review of district hospital records showed that national figures underestimated the incidence of snake bite by one order of magnitude [12]. The highest figures reported in Asia so far come from a community-based survey conducted in southeast Nepal in 2002, which revealed annual incidence and mortality rates of 1,162/100,000 and 162/100,000, respectively [13]. Figures of a similar magnitude were recently also obtained in a nation-wide community-based survey in Bangladesh (M. R. Rahman, personal communication).

Snake bite is an important occupational injury affecting farmers, plantation workers, herders, and fishermen. Open-style habitation and the practice of sleeping on the floor also expose people to bites from nocturnal snakes. As summarized in Table 1, several epidemiological studies have outlined characteristics of snake bite victims in the region. Bites are more frequent in young men, and generally occur on lower limbs. The incidence of snake bites is higher during the rainy season and during periods of intense agricultural activity [14],[15]. Snake bite incidence and mortality also increase sharply during extreme weather events such as floods. During the 2007 monsoon flood disaster in Bangladesh, snake bite was the second most common cause of death, after drowning, eclipsing mortality from diarrheal and respiratory diseases and illustrating how important snake bite can be in this region compared to other health problems [16].

**Table 1:** Characteristics of snake bite victims in South Asia.

Characteristic	Details	References
**Age**	The mean age of snake bite victims is around 30 years. Three-quarters of the victims are in the 10- to 40-year age group, broadly in agreement with demography.	[7], [9], [12], [14], [15], [22], [37], [40], [49], [55], [56], [76], [88], [89], [93], [94], [97], [104], [111]–[119]
**Gender**	There is a clear preponderance of males among snake bite victims. A 2∶1 male to female ratio is frequently observed.	[7], [9], [12], [14], [15], [22], [37], [40], [49], [55], [56], [76], [88], [89], [93], [94], [97], [104], [111]–[119]
**Occupation**	Farmers account for more than half of the victims. Students and housewives are also frequently bitten.	[9],[13],[15],[22],[40],[56],[93],[114],[117],[120]
**Time of bite**	The time of bite depends on the relative abundance of diurnal and nocturnal snakes. Krait bites generally occur at night, whereas viper and cobra bites mostly occur during daytime.	[7], [9], [12], [14], [15], [22], [37], [49], [56], [88], [89], [94], [97], [104], [114]–[117]
**Site of bite**	60%–80% of bites occur on the foot, ankle, or leg. Bites on the head and trunk are mostly due to nocturnal species biting sleeping people.	[7], [9], [12], [14], [15], [22], [40], [49], [56], [76], [77], [88], [93], [97], [104], [114]–[118],[121],[122]
**Delay between bite and treatment**	The bite-to-treatment delay varies greatly, ranging from 30 minutes to 15 days. Most studies show that at least 60% of victims reach a health centre within six hours but very few in less than one hour.	[7],[12],[14],[15],[22],[40],[49],[55],[56],[74],[88],[93],[94],[104],[113]–[119],[121]
**First aid methods**	In eight out of 15 studies, more than 50% of snake bite victims used inappropriate and harmful first aid methods. Tourniquets are used by up to 98% of patients.	[7], [9], [13], [22], [40], [49], [74], [76], [88], [93], [104], [113], [115]–[117],[119]}
**Mortality**	Mortality rates are highly variable, ranging from 0.5% to 58%. Most fatalities occur before reaching treatment centres.	[7], [9], [12]–[15], [22], [37], [49], [55], [56], [76], [88], [89], [93], [94], [97], [104], [111], [113]–[121]

## Venomous Snakes in South Asia

The number of different snake species found south of the Himalayas is estimated to be around 300, including about 67 front-fanged venomous species of the families Elapidae and Viperidae [17]–[21].

Viperid snakes are represented by 26 species belonging to the true vipers (subfamily Viperinae) and pit vipers (Crotalinae). Among the true vipers, Russell's viper (*Daboia russelii*) is associated with the highest morbidity and mortality. In Anuradhapura District, Sri Lanka, up to 73% of all admitted snake bites are attributed to this species [22] whose distribution extends north to the Indus valley of Pakistan and Kashmir, to the foothills of the Himalayas in Nepal and Bhutan and to Bangladesh in the east. Saw-scaled vipers (*Echis carinatus* and *E. sochureki*) are other very important viperine species that inhabit open and dry environments. *E. sochureki* causes numerous bites in northern India and has long been regarded as one of Pakistan's deadliest snakes [17],[23]; *E. carinatus* is regionally highly abundant and causes many bites in parts of western and southern India [18] and in arid coastal areas of northern Sri Lanka [24]. Three other species of true vipers that occur in the west of South Asia are the Levantine viper (*Macrovipera lebetina*) and two species of desert vipers (*Eristicophis macmahoni* and *Pseudocerastes persicus*). Although their bites have been considered to be comparatively rare, they are capable of causing severe envenoming [25],[26].

Pit vipers belonging to various genera [27],[28] have traditionally been regarded as being of lesser concern in South Asia. However, these snakes occur in most habitat types from mangroves to the high mountains, and some species are common in gardens and agricultural landscapes. Envenoming by green pit vipers is very common in wide regions of Bangladesh and Nepal [12],[29], and bites by the mountain pit viper (*Ovophis monticola*) occur in Nepal where it is the most frequently encountered venomous snake at altitudes of 900–2,700 m [21],[30]. Although causing few fatalities, bites by these species produce marked local effects that can result in chronic conditions and permanent sequelae [31],[32]. In southern India, recent studies have reported massive morbidity among plantation workers due to bites by a much smaller species, the Malabar pit viper (*Trimeresurus malabaricus*) [33]. Hump-nosed pit vipers (*Hypnale hypnale* and *H. nepa*) are also emerging as medically important species in the region, and can cause renal failure and haemostatic dysfunctions [34],[35]. Several fatalities due to *H. hypnale* envenoming, for which there is no specific antivenom, were reported in India and Sri Lanka [34],[36],[37].

The family Elapidae is represented by at least 17 terrestrial species (including cobras, king cobras, kraits, and coral snakes) and numerous species of sea snakes in South Asia. Bites by cobras (*Naja* species), which are best known for raising their head and anterior body and spreading their neck as a hood in defence, typically occur outdoors in the late afternoon [17],[18]. The spectacled cobra (*Naja naja*), one of India's commonest snakes, causes numerous cases of envenoming every year [38]. In the northern and eastern parts of the Indian subcontinent, the monocellate cobra (*N. kaouthia*) also belongs to the medically important snakes. A third cobra species, *N. oxiana*, occurs in the northwest [18],[20],[21]. Kraits (*Bungarus* species) are slender, nocturnal snakes that often enter human dwellings at night in search of prey. Consequently, many victims of krait bites are bitten while asleep. Case fatality rates of krait envenoming reach up to 77%–100% without treatment [17],[39]. Traditionally, most krait bites in South Asia have been attributed to the common krait (*Bungarus caeruleus*), however, in South Asia alone there are eight species of *Bungarus*, several of which are morphologically similar to *B. caeruleus*. Several studies have demonstrated that a number of these are medically important in the region [40]–[43]. Coral snakes are smaller elapid snake species that are brightly coloured. They are rarely encountered by humans and thus are thought to cause few bites, but fatalities have been reported [44]. Sea snakes are large, paddle-tailed and primarily marine snakes that feed on fish and fish eggs. They are often caught in the nets of fishermen who are at risk of bites while handling them. Most fatalities following sea snake bites are attributed to *Enhydrina schistosa* [45] but the species identity of sea snakes involved in bites in South Asia has only very rarely been established [46],[47].

An often overlooked problem is that of nonvenomous or mildly venomous species. They represent the vast majority of living snakes, and may be mistaken for venomous snakes and/or involved in snake bite in South Asia. Rat snakes (*Ptyas* species, *Coelognathus* species) are large, rapidly moving snakes that are often confused with cobras. Most notoriously, several genera of small nonvenomous snakes share the same colour pattern as kraits [19],[48]. Wolf snakes (*Lycodon* species) are of particular concern in this regard because some of them (e.g., *Lycodon aulicus*) are very common inside and around houses and bite aggressively if disturbed [40].

This species diversity has a significant public health impact: in addition to increasing the risk of bites in all kinds of environments, it complicates clinical management with respect to both diagnosis and treatment, as well as antivenom design and manufacture and control strategies.

## Clinical Features of Snake Bite Envenoming

A widespread belief is that snake bites inevitably result in envenoming. However, bites by nonvenomous snakes are common and bites by venomous species are not always accompanied by the injection of venom (dry bites). A large survey conducted in ten hospitals of southern Nepal revealed that envenoming occurred in only 10% of the victims [12]. In Kerala, India, only 219 out of 635 patients (34%) with proven snake bite developed signs of systemic envenoming [49]. Likewise, in Bangladesh the proportion of nonenvenomed bites reported in hospital-based studies varied between 60% and 80% [29],[50]. Moreover, as symptoms associated with panic or stress sometimes mimic early envenoming symptoms, clinicians may have difficulties in determining whether envenoming occurred or not.

**Figure 1:**
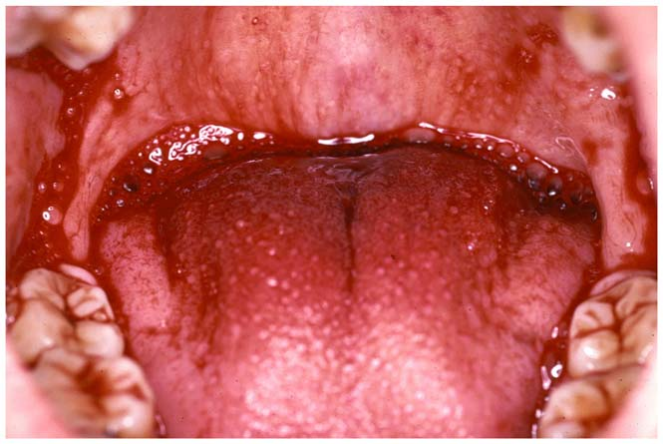
Gum bleeding after bite by Russell's viper. In Asia, coagulation defects and spontaneous bleeding are characteristic of bites by viperid snakes and are caused by procoagulant and haemorrhagic toxins in the snake venom. *Image credit: D. A. Warrell*.

When envenoming does occur, it can be rapidly life-threatening. Snake venom is a complex mixture of toxins and enzymes, each of which may be responsible for one or more distinct toxic actions. In bites by South Asian viperid snakes, envenoming results in local pain and tissue damage, characterised by swelling, blistering, bleeding, and necrosis at the bite site, sometimes extending to the whole limb [17]. Viperid venoms can also induce coagulopathy and platelet dysfunction, leading to spontaneous systemic haemorrhages and persistent bleeding from fang marks, wounds, or gums (Figure 1). Intracranial bleeding, including anterior pituitary haemorrhage, and multi-organ failure are common causes of death [51]. A prospective study conducted in Anuradhapura District, Sri Lanka, showed that 92% of patients with Russell's viper envenoming presented with local swelling and 77% had haemostatic disturbances [7]. In addition, Russell's viper can cause acute renal failure and neurotoxicity, as has been shown in several studies conducted in south India and Sri Lanka [7],[22],[49],[52].

**Figure 2:**
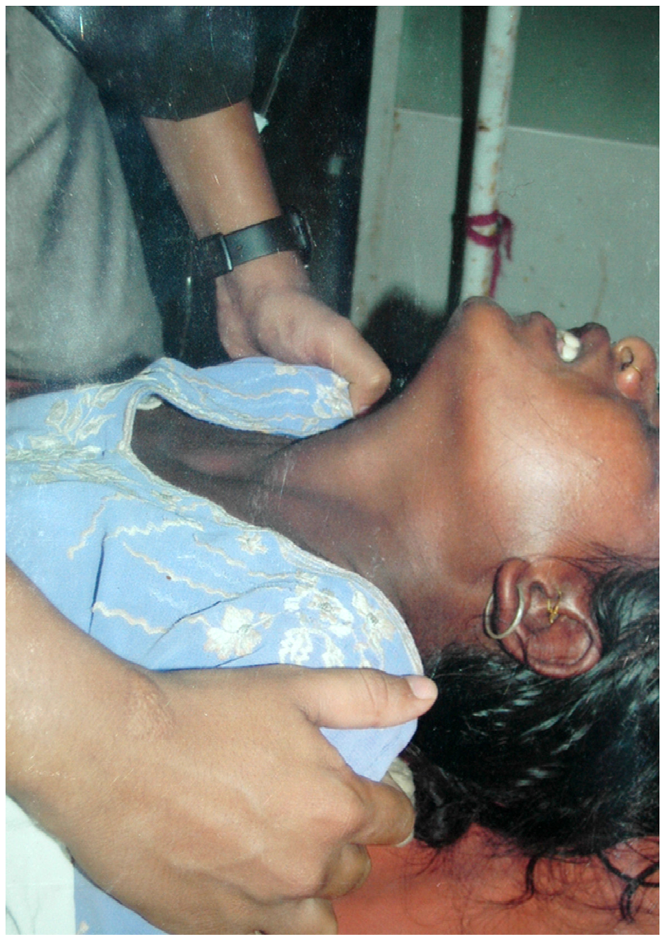
“Broken neck” sign observed in a 14-year-old girl bitten by a Russell's viper in India. Envenoming by cobras, kraits and—in some areas—by Russell's viper frequently leads to progressive descending paralysis. Looking for the broken neck sign, which is caused by paralysis of the neck flexor muscles, should be part of the routine clinical assessment of patients. In this case, neuroparalysis persisted for five days despite antivenom treatment, but without progression toward respiratory failure. *Image credit: H. S. Bawaskar*.

Among the Elapidae, bites by *N. naja* and *N. kaouthia* can cause significant local swelling and sometimes extensive tissue necrosis of the bitten limb [31],[38],[53], whereas bites by kraits or sea snakes do not usually cause signs of local envenoming and can be virtually painless. Cobra venom contains mainly postsynaptic neurotoxins, which bind and block acetylcholine receptors of the neuromuscular junction, while krait venom in addition contains presynaptic toxins that damage nerve endings [54]. Progressive descending paralysis is the hallmark of systemic envenoming by elapid snakes in South Asia (Figure 2). Extraocular muscles are particularly sensitive to neuromuscular blockade, leading to a droop of upper eyelids (bilateral ptosis), a frequently observed early sign of paralysis [55]. Patients are often unable to protrude their tongue beyond the incisors and may present with difficulty speaking or swallowing. Limb weakness, loss of deep tendon reflexes, and fixed dilated pupils may follow. Once paralysis reaches the diaphragm and the intercostal muscles, victims usually die of respiratory failure if they are not adequately ventilated. Hospital-based studies in Sri Lanka showed that 48%–64% of *B. caeruleus* victims developed respiratory paralysis and required mechanical ventilation [15],[56]. Although many clinical signs of neurotoxic envenoming by cobras and kraits are similar, with both genera able to cause respiratory failure within 30 minutes of the bite [57],[58], krait bite envenoming is often associated with a delayed onset and prolonged total period of paralysis. This is due to the function and effects of the most lethal components of krait venoms, beta-bungarotoxins, which destroy nerve terminals [59]. While coagulopathy and bleeding are common features of envenoming by certain elapid snakes in Australia and New Guinea [60], clinical bleeding and clotting problems have not been reported after bites by South Asian elapids. Likewise, systemic myotoxicity following elapid snake bites was previously known only from sea snakes and some of their terrestrial relatives in Australia and New Guinea [60]. However, venom-induced generalized rhabdomyolysis and renal failure has recently also been observed in envenoming by the greater black krait (*B. niger*) in Bangladesh, further complicating clinical management (Faiz et al., unpublished data).

## Diagnosis

The identification of snake species is crucial for optimal clinical management, because it allows clinicians to choose the appropriate treatment, anticipate complications, and therefore to improve prognosis. Moreover, as specific antivenoms are not available for South Asian pit vipers and most krait species, identifying these species would help to avoid wasting this expensive treatment and exposing patients to antivenom-induced adverse reactions. As mentioned above, bites by nonvenomous species are common and may throw clinicians into confusion.

Unfortunately, in many cases the biting snake is not seen, and if it is, its description by the victim is often misleading [40]. Even when the dead snake is brought to the health centre, misidentification is common. For example, hump-nosed pit vipers (*H. hypnale*) are frequently misidentified as saw-scale vipers (*E. carinatus*) in Kerala, India [34]. Consequently, many *H. hypnale* bite victims end up receiving ineffective antivenom. Throughout South Asia, krait bites are routinely attributed to *B. caeruleus* based on clinical syndromes and the great superficial similarity of *B. caeruleus*, *B. sindanus*, and *B. walli*. However, envenoming by krait species other than *B. caeruleus* that does not respond to available antivenoms may be common, as observed in Bangladesh (Faiz et al., unpublished data; Kuch et al., unpublished data).

Most physicians in South Asia have to rely on the circumstances of the bite and the clinical features of envenoming to infer the biting species. Coagulopathy, when present, is diagnostic of viper and pit viper bites in South Asia and can be observed using the 20-minute whole blood clotting test [61]. In Sri Lanka, *B. caeruleus* envenoming has a characteristic epidemiologic and clinical pattern [15]. Syndromic approaches have been proposed to assist physicians in identifying the biting species [61] and attempts have been made to develop clinical scores based on envenoming features [62]. However, careful studies of envenoming profiles are lacking for most species in this region. Thus, more clinical research using reliably identified snakes is needed to further explore differences in envenoming syndromes between additional species, and to evaluate their applied utility.

Immunoassays for detecting venom antigens in body fluids have been described for a number of species [63]–[65], and attempts have been made to develop ELISA tests for South Asia [66]–[68]. Unfortunately, a narrow focus on an insufficient number of species and cross-reactivity between venoms has so far hindered the development of a reliable diagnostic test [64],[69]. To limit the problem of cross-reactivity, the use of purified species-specific toxins for immunization or affinity-purified venom-specific polyclonal antibodies may be worth considering [70]. However, even then the paucity of reliable data on the diversity and distribution of venomous snakes in South Asia, the unavailability of venom for almost all of the species, and the very limited insight into venom variability even within the commonest species—all caused or promoted by restrictive wildlife legislation—remain major obstacles for the design and production of immunodiagnostics. Under these aspects, the use of forensic molecular techniques as an explorative and complementary tool for snake species diagnosis is promising. Forensic routine has shown that it is feasible to identify an aggressor (e.g., human or dog) based on trace DNA from bite marks [71], and this is also possible in the case of snakes [72]. PCR amplification and sequencing of snake DNA obtained from bite-site swabs has recently been used to identify biting snakes in an animal model and in clinical cases from Bangladesh and Nepal (Kuch et al., unpublished data). The utility of this method as a clinical diagnostic tool, however, awaits further study.

## Management of Snake Bite Victims and Recommended Treatment

Health workers in rural districts are usually poorly trained to manage snake bite envenoming, which is a complex emergency. A recent survey conducted in India and Pakistan showed that many doctors were unable to recognize systemic signs of envenoming [73]. Another study in northwest India revealed that most snake bite victims presenting at primary health centres received inadequate doses of antivenom and that out of 42 patients who required assisted ventilation, only one was intubated [74]. Improving the knowledge of care-givers at all levels of the health system is a challenge of paramount importance and great urgency in South Asia. Papua New Guinea, where snake bite management training programmes have been implemented in both rural and urban hospitals, could serve as an inspiring model in this regard.

### First aid

Most experts agree that snake bite victims should be transported as quickly as possible to a medical centre where they can be clinically evaluated by qualified medical staff, and where antivenoms are available. In fact, time of transport was shown to be a crucial determinant of snake bite mortality in eastern Nepal [13], and studies in southern India confirmed that delayed antivenom administration was associated with an increased risk of complications [49],[75]. The bite victim should be reassured, the bitten limb immobilized with a makeshift splint or sling, and the patient transported. Walking is contraindicated, because muscular contractions promote venom absorption.

**Figure 3:**
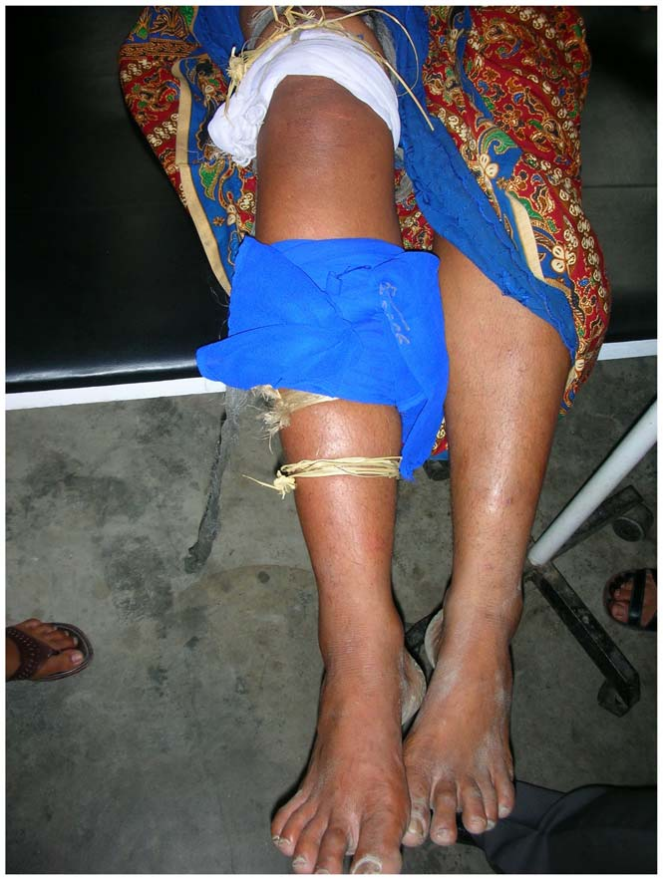
Tourniquet on a 43-year-old woman presenting at a rural health post in Nepal. First-aid methods applied throughout South Asia are largely inadequate. Tourniquets in particular can have deleterious effects. This patient consulted after being bitten by a nonvenomous rat snake (*Ptyas mucosa*) that she had killed and brought for identification. After reassurance, local treatment, and observation, she was uneventfully discharged from the health post. *Image credit: E. Alirol*.

These simple recommendations are unfortunately rarely followed and vital time is often lost. The majority of victims first report to traditional healers [10],[40],[55],[76]. Popular traditional treatments include chanting, incisions, attempts to suck venom from the bite site, and the application of herbal medicine or snake stones. Two studies in Nepal and Bangladesh showed that 90% and 98% of snake bite victims, respectively, used tourniquets (Figure 3) [40],[77]. In Bangladesh, incisions at and around the bite site were made in 28% of envenomed victims and in 13%–14% of those without signs of envenoming [40]. In northwest India, incision and drainage were practiced by 20% of patients [74]. These traditional measures are strongly contraindicated as they are ineffective and in most cases deleterious. For example, tourniquets cannot be safely left on for long without risking severe local damage including ischemia, necrosis, and gangrene [78],[79].

In 1979, Sutherland et al. recommended the pressure-immobilization method as an effective alternative first aid method [80]. According to the authors, the bitten limb should be bound firmly with a crepe bandage, starting distally around the toes or fingers and moving proximally. Although this technique has been extensively promoted in Australia, its efficacy remains controversial [81]. For example, a recent study in Australia showed that crepe bandages rarely generated optimal pressures compared with elasticized bandages [82]. In a study in India, pressure-immobilization was found to be difficult to apply correctly despite intense previous training of care providers [83]. In the Australian study, training did improve participants' ability to apply elasticized bandages [82], and in a study in Papua New Guinea, participants living in an area where snake bites are common were very successful in gaining and retaining the ability of correctly applying pressure-immobilization (D. Williams, personal communication). This method is, however, contraindicated for viper and cobra bites as it may increase local tissue damage [81],[84] and may contribute to delaying transport of the victim to a treatment centre.

### Antivenoms

Immunotherapy is the only specific treatment for snake bite envenoming. Antivenoms are produced by fractionation of plasma obtained from immunized animals, usually horses [85]. They can be either monovalent or polyvalent, depending on the number of species (single or multiple, respectively) whose venoms are used for immunization. Although monovalent antivenom has often been considered more efficacious, the production of polyvalent antivenom is preferred in many countries as snake species identification is generally not possible for the attending physician. Antivenoms have been available in South Asia for the past 60 years, and all existing products are manufactured by Indian companies. Traditionally, the production has focused on four species believed to be responsible for most deaths: *N. naja*, *B. caeruleus*, *D. russelii*, and *E. carinatus*. However, a number of other species that contribute to morbidity and mortality in the region have not been considered, and envenoming by these species usually does not respond adequately to existing antivenoms [34],[36].

The success of antivenom therapy depends on the ability of immunoglobulins to bind, extract, and eliminate toxins present in the body. While their efficacy in restoring haemostasis and cardiovascular functions is well established, the ability of antivenoms to prevent tissue damage and to reverse neurotoxicity is more controversial [56],[86],[87]. For instance, administration of antivenom to krait bite victims with established respiratory paralysis does not reverse paralysis [55],[86],[87]. This lack of clinical effectiveness often contributes to the administration of excessive amounts of antivenom [88],[89]. Moreover, treatment outcome can vary greatly with the geographical area as the venom composition and antigenic properties of toxins can be highly variable across the range of a given snake species [90],[91]. Indian antivenoms are produced using venoms from snakes captured in a tiny geographic area of the State of Tamil Nadu, and may therefore be less effective in other regions [92]. For example, the efficacy of Indian polyvalent antivenoms for the treatment of envenoming by Russell's viper in Sri Lanka is controversial [22],[93].

As a matter of fact, most of the antivenoms that are routinely used in South Asia have never been subjected to independent preclinical testing and formal evaluation in clinical trials. Their efficacy and safety profiles have not been properly established, and there is currently no evidence-based protocol for their administration and dosage. Up to 80% of patients treated with Indian antivenoms present one or more adverse effect(s) such as anaphylactoid or pyrogenic reactions, or late serum sickness [37],[93],[94]. While to our knowledge no fatal cases have been reported in South Asia, severe drug reactions occur and are likely to be under-reported. Adverse reactions can be efficiently managed by cheap, widely available drugs (e.g. antihistaminics, corticoids, adrenalin), but their prophylactic use yielded contradictory results [94]–[96]. The risk of severe adverse events exists but must be balanced against the life-saving potential of this treatment.

Antivenoms may be supplied free of cost by some ministries of health but their supply remains insufficient and irregular in several countries [12], leading to the purchase of drugs by the patients' relatives. One vial of antivenom of Indian production costs around US$8–10, which is equivalent to several days of salary for poor farmers. Thus, many cannot afford to purchase the average 10–15 vials needed to reverse envenoming [97].

### Ancillary treatment

The management of envenomed snake bites is not limited to the administration of antivenoms. In the case of neurotoxic envenoming, artificial ventilation and careful airway management are crucial to avoid asphyxiation in patients with respiratory paralysis. Cases of complete recovery from severe neuromuscular paralysis without antivenom have been reported after prolonged artificial ventilation [98].

Anticholinesterase drugs such as edrophonium can partly overcome blockade by postsynaptic neurotoxins and have shown good efficacy in cobra bite envenoming [61],[99]. A few cases of successful anticholinesterase use have also been reported in krait bite envenoming in India [100], but there is currently no treatment to stop the destruction of nerve endings by presynaptic krait toxins once this degeneration process has started.

Bacterial infections can develop at the bite site, especially if the wound has been incised or tampered with nonsterile instruments, and may require antibiotic treatment. However, there are currently no data supporting their systematic use [101]. A booster dose of tetanus toxoid should be administered but only in the absence of coagulopathy [17]. Necrosis on the bitten limb may require surgery and skin grafts, particularly in the case of cobra bites. If necrotic tissues are not removed, secondary bacterial infections can occur [53]. Tensed swelling, pale and cold skin with severe pain may suggest increased intracompartmental pressure in the affected limb. However, fasciotomy is rarely justified. In particular, it can be disastrous when performed before coagulation has been restored. A clear proof of significant compartment syndrome by measurement of substantially elevated intracompartmental pressures is a prerequisite [61].

## Control and Prevention

In practice, strategies to control snake populations and to prevent snake bites are nonexistent in South Asian countries. Many bites could be avoided by educating the population at risk. Sleeping on a cot (rather than on the floor) and under bed nets decreases the risk of nocturnal bites in Nepal [55],[102]. Rubbish, termite mounds, and firewood, which attract snakes, can be removed from the vicinity of human dwellings. Attempts can be made to prevent the proliferation of rodents in the domestic and peridomestic area. Thatched roofs, and mud and straw walls are favoured hiding places for snakes and should be checked frequently. Many bites occur when people walking barefoot or wearing only sandals accidentally step on a snake. Using a torch/flashlight while walking on footpaths at night, and wearing boots [103] and long trousers during agricultural activities, could significantly reduce the incidence of bites.

A complementary strategy is to decrease the risk of dying from envenoming snake bites. Many areas where snake bite envenoming occurs are relatively inaccessible by road, especially during the rainy season, and transport to a health centre sometimes takes more than 24 hours [14],[74],[104]. In Nepal, a programme for rapid transport of snake bite victims by motorcycle volunteers to a specialized treatment centre significantly reduced the risk of fatal outcome (Sharma et al., manuscript in preparation).

## Conclusion

While sub-Saharan Africa faces a dramatic crisis in antivenom production and supply [105],[106], shortage of antivenom is not the most pressing issue in South Asia. Indeed, it is estimated that India produces around one million vials of antivenom each year [107]. Despite these large volumes of production, several challenges persist that prevent appropriate management of snake bite victims in South Asia. Poor access to often inadequately equipped and staffed medical centres in rural areas, high cost of treatment, and inadequate use of antivenoms are major concerns [108],[109]. Increased attention and means should be dedicated to snake bite envenoming by researchers, funding agencies, pharmaceutical industries, public health authorities, and supranational organisations, as all have contributed to keeping this important public health problem a truly neglected disease.

**Box 1. Key Learning Points**

- South Asia has the highest incidence and mortality rates of snake bite in the world.
- Bites by venomous snakes in this region can cause local tissue damage, neuroparalysis, systemic haemorrhages, generalized myotoxicity, acute renal failure, or complex combinations of these.
- Recommended first aid measures include reassurance of the snake bite victim, immobilization of the bitten limb, and rapid transport to a competent treatment centre.
- Antivenom is the only specific treatment for snake bite envenoming, but existing products cover only a very limited number of medically significant species.

**Box 2. Five Key Papers in the Field**

1. Kasturiratne A, Wickremasinghe AR, de Silva N, Gunawardena NK, Pathmeswaran A, et al. (2008) The global burden of snakebite: A literature analysis and modelling based on regional estimates of envenoming and deaths. PLoS Med 5: e218. doi:10.1371/journal.pmed.0050218
2. Warrell DA (1999) WHO Guidelines for the clinical management of snake bites in the South East Asia Region. SE Asian J Trop Med Publ Health 30: 1–83.
3. World Health Organization (2007) Rabies and envenomings: A neglected public health issue. Geneva: WHO. Available at http://www.who.int/bloodproducts/animal_sera/rabies_envenomings/en/index.html
4. Ariaratnam CA, Thuraisingam V, Kularatne SA, Sheriff MH, Theakston RD, et al. (2008) Frequent and potentially fatal envenoming by hump-nosed pit vipers (*Hypnale hypnale* and *H. nepa*) in Sri Lanka: Lack of effective antivenom. Trans R Soc Trop Med Hyg 102: 1120–1126.
5. Gutiérrez JM, Theakston RDG, Warrell DA (2006) Confronting the neglected problem of snake bite envenoming: The need for a global partnership. PLoS Med 3: e150. doi:10.1371/journal.pmed.0030150

**Box 3: Main Challenges**

(1) Improving access

Access to care is hindered both by the remoteness of snake bite–prone areas and by the cost of snake bite management. A community survey in Nepal showed that snake bite envenoming represents a substantial financial burden for rural households [13]. Despite the mushrooming of well-equipped private clinics in some rural areas of India, poor villagers rarely have access to mechanical ventilation or dialysis.

(2) Improving clinical management

Simple and standardized protocols on snake bite management are needed. Despite the publication of regional guiding principles [61], national protocols are not always consistent with each other (e.g., low initial dose of antivenom advised in Nepal versus high initial dose in India and Bangladesh), are often not available in peripheral health structures and are poorly explained to end users. Moreover, manufacturers' recommendations are often misleading [107]. In Nepal, the application of different protocols may play a role in the wide range (3%–58%) of case-fatality rates reported from various hospitals [12].

(3) Improving diagnostic and treatment tools

The lack of field-applicable diagnostic tools to identify snake species contributes to poor case definitions [110], mismanagement of patients, and uncertainties about snake bite epidemiology. Snake bite victims in South Asia are still reliant on old generations of antivenoms, and several venomous species are not covered by existing products [92]. The pharmacokinetic and pharmacodynamic properties, efficacy, and safety of most Indian antivenoms have never been studied or compared. WHO has recently endorsed the strengthening of antivenom production, and efforts are being made to help Indian manufacturers to improve the quality of existing products [108]. However, the impact of this approach on the cost of antivenom production needs to be carefully anticipated and closely monitored [1].

(4) Improving knowledge

Improving the knowledge of both care-givers and rural communities is crucial. Health workers in rural districts are usually poorly trained to deal with this complex emergency. For example, many doctors in India and Pakistan appear to be unaware of the criteria for antivenom administration [73]. Education of rural communities on snake bite, avoidance of useless or dangerous first-aid measures, and the importance of rapid transport of victims to treatment centres should be widely implemented [13].
